# Macrophage–Fibroblast Crosstalk in Kidney Injury: A Narrative Review

**DOI:** 10.3390/ijms27188173

**Published:** 2026-09-14

**Authors:** Panpan Qiang, Lizheng Han, Ruyan Lv, Zhenfei Dong, Teng Ma, Xiangting Wang, Qingyou Xu

**Affiliations:** 1 College of Integrated Traditional Chinese and Western Medicine, Hebei University of Chinese Medicine, Shijiazhuang 050200, China; 2 Hebei Key Laboratory of Integrative Medicine on Liver-Kidney Patterns, Hebei University of Chinese Medicine, Shijiazhuang 050091, China

**Keywords:** fibroblasts, macrophages, cell crosstalk, kidney

## Abstract

Renal fibrosis is a multifaceted pathological process driven by cellular interactions, with fibroblasts and macrophages serving as central regulators. Fibroblasts activate into matrix-producing myofibroblasts, directly contributing to fibrogenesis and disease progression. Macrophages, characterized by pronounced heterogeneity, dynamically modulate the balance between tissue injury and repair, thereby critically influencing disease outcomes. The crosstalk between these two cell types operates through diverse signaling pathways, extracellular vesicle-mediated communication, and metabolic reprogramming, forming an intricate regulatory network that exerts stage-specific effects during renal injury. Recent insights into fibroblast activation, macrophage polarization, and their intercellular interplay have provided potential therapeutic avenues for delaying kidney fibrosis, offering renewed promise for clinical translation.

## 1. Introduction

The kidney is essential for systemic homeostasis, regulating water, electrolyte, and acid–base balance via glomerular filtration and tubular transport to maintain stable osmotic pressure, blood volume, and pH. Clinically, kidney injury is categorized by disease course into acute kidney injury (AKI) and chronic kidney disease (CKD). AKI is a syndrome of rapid renal function decline, often from ischemia–reperfusion, sepsis, or nephrotoxins; CKD involves progressive, irreversible structural and functional loss, typically from diabetes, hypertension, or glomerulonephritis, with glomerulosclerosis and interstitial fibrosis as key features. Although some patients with AKI could recover fully, epidemiological evidence indicates that AKI is not merely an isolated acute event but also a significant risk factor for CKD [1,2]. Both AKI and CKD involve complex pathological alterations, including inflammation, microvascular rarefaction, metabolic reprogramming, and progressive extracellular matrix (ECM) deposition, among which aberrant crosstalk between immune cells and stromal cells is considered a central driver of fibrotic progression [3,4].

The normal structure and function of the kidney rely on the interplay and dynamic equilibrium between cells and the ECM. The ECM not only serves as a physical scaffold for tissue but also regulates cell proliferation, differentiation, and biological functions through biomechanical signaling, constituting the center of intercellular communication [5]. As the prominent resident innate immune cells in the kidney, macrophages exhibit plasticity, dynamically transitioning from pro-inflammatory to repair-oriented or pro-fibrotic phenotypes at different stages of injury, and modulating ECM remodeling through the secretion of various cytokines. Fibroblasts are the dominant ECM-producing cells; during injury, they activate into α-smooth muscle actin (α-SMA)-positive myofibroblasts that deposit excessive ECM, thereby directly driving fibrotic progression. In recent years, advances in Single-cell RNA sequencing (scRNA-seq) and spatial transcriptomics have provided unprecedented perspectives for analyzing the heterogeneity and crosstalk of these two cell types within the renal microenvironment.

The interaction between macrophages and fibroblasts constitutes a multi-pathway, dynamically regulated network involving diverse modes of crosstalk. In the acute phase of AKI, macrophages first assume an M1-like phenotype to clear pathogens and dead cells and promote inflammation, then transition toward a repair phenotype that modulates fibroblast activity [6,7]; excessive fibroblast activation, however, leads to ECM deposition and fibrosis [7]. In CKD, macrophages persist in a mixed activation state and further stimulate fibroblasts via TGF-β and other pathways, thereby accelerating disease progression [8]. Notably, this crosstalk is not unidirectional. Macrophage-to-myofibroblast transition (MMT) has attracted much attention as it contributes directly to ECM synthesis, but the relative contribution and mechanism in vivo remain controversial [9,10]. Conversely, fibroblasts reciprocally regulate macrophage recruitment and polarization through secreted factors such as CCL2 and CSF1, establishing a feedback loop that sustains injury progression [9,10].

In recent years, the critical role of bidirectional crosstalk between macrophages and fibroblasts in tissue injury repair and fibrotic progression has garnered increasing attention, accompanied by a growing body of research. Despite notable advances, existing reviews acknowledge that this field still faces considerable challenges. On one hand, the specific signaling pathways and key molecules involved in macrophage–fibroblast crosstalk during kidney injury have yet to be systematically delineated. On the other hand, a comprehensive overview of how their interaction patterns differ across different disease stages of renal injury remains lacking. Therefore, a review of the current state of macrophage–fibroblast crosstalk in kidney injury is of significant theoretical and clinical value for deepening our understanding of renal injury mechanisms and identifying novel therapeutic targets.

It should be noted that, as a narrative review, this article does not aim to provide a comprehensive systematic search and quality assessment of all studies in the field of renal fibrosis. Rather, it focuses on the specific area of macrophage–fibroblast crosstalk. Specifically, this review intends to systematically review the molecular mechanisms and pathological significance of their interaction in kidney injury, with special attention to: (1) the heterogeneity and spatial distribution characteristics of macrophages and fibroblasts; (2) the key signaling pathways and molecular networks mediating their crosstalk; (3) the dynamic evolution of this interaction during AKI and CKD; and (4) potential therapeutic strategies targeting this crosstalk. A thorough understanding of macrophage–fibroblast interaction holds significant scientific and clinical implications for elucidating the pathogenesis of renal fibrosis, identifying novel biomarkers and therapeutic targets. On this basis, we propose a conceptual framework and summarize future research directions.

## 2. Macrophages and Fibroblasts in Kidney Injury

### 2.1. Macrophages in Kidney Injury

The renal macrophage comprises tissue-resident macrophages and circulating monocyte-derived macrophages. These populations differ in ontogeny, mechanisms of homeostatic maintenance, and injury responses. Lineage-tracing studies indicate that most resident macrophages in the mouse kidney arise from fetal liver monocytes, expand during postnatal kidney development, and persist into adulthood through local self-renewal. Under steady-state conditions, bone marrow-derived monocytes (BMDM) could enter the kidney and replenish part of the macrophage pool after depletion or tissue injury. In mice, resident renal macrophages are typically F4/80^hi^ CD11b^low^ and are enriched for *C1qc*, *Cx3cr1*, *CD74*, and *CD81*, whereas recruited monocyte-derived macrophages are more commonly CD11b^hi^ F4/80^low^ and often express *Ly6C* and *CCR2* during the early phase of recruitment [11,12,13]. Cross-species scRNA-seq further suggests that *CD74* and *CD81* are relatively conserved features of resident renal macrophages across humans, mouse, pig, and rat. Nevertheless, the alignment of macrophage characteristics across species is primarily based on transcriptomic similarity and should not be taken as evidence of functional equivalence [13].

Renal macrophages exhibit significant heterogeneity in both spatial distribution and function. In the healthy kidney, these cells are regionally distributed along the cortex, medulla, and papilla. scRNA-seq and spatial transcriptomics have identified multiple subpopulations, including heme/iron metabolism-associated macrophages, medullary CD14^+^ inflammatory macrophages, type I interferon-responsive macrophages, and fibrosis-associated macrophages, identified that transcriptional identity and function are spatially constrained [14,15]. Following ischemia–reperfusion injury (IRI), subpopulations normally confined to the medulla and papilla appear at the corticomedullary junction and cortex in the early phase; however, it remains unclear whether this results from cell migration or transcriptional changes. By day 28, the heme/iron metabolism-associated subpopulation has largely regained its original distribution, whereas the inflammatory, fibrosis-associated, and type I interferon-responsive subpopulations remain scattered, indicating that ischemic injury can durably remodel the spatial niches of resident macrophages [15].

Macrophage activation has traditionally been described as M1/M2, particularly in in vitro experimental systems. In murine systems, classical activation (M1) induced by LPS and IFN-γ is generally associated with increased Nos2/iNOS expression and the production of inflammatory mediators such as TNF-α, IL-1β, and IL-6, whereas IL-4 or IL-13 induces an alternative activation (M2) characterized by Arg1 and CD206 [16,17]. Transcriptomic studies, however, have demonstrated that macrophage activation spans a stimulus-dependent, multidimensional continuum rather than two discrete endpoints [17,18]. The advent of scRNA-seq and spatial transcriptomics has further advanced the classic M1/M2 binary classification and deepened understanding of macrophage heterogeneity in fibrotic kidney.

Recruited monocyte-derived macrophages tend to exhibit a pro-inflammatory phenotype. In mouse ischemia–reperfusion injury (IRI), the monocyte-derived S100a9^hi^Ly6c^hi^ subset accumulates rapidly during the early injury, and contributes to inflammatory amplification [19]. In models of AKI-to-CKD, a proinflammatory macrophage population expressing *Il1b*, *Tnf*, *Ccl3*, *Ccl4*, *Cxcl2*, *Cxcl10*, and *Ccl12* has also been identified. Its transcriptional profile is mainly associated with acute inflammatory responses and chemotaxis [20]. In human ANCA-associated glomerulonephritis, monocyte-derived macrophages express a chemotactic gene set comprising *CXCL2*, *CXCL3*, *CXCL8*, and *CCL3*, together with the proinflammatory genes *IL1β* and *TNF* [21]. Persistent injury can drive recruited macrophages toward ECM-remodeling or profibrotic states. In the unilateral ureteral obstruction (UUO) model, early recruited Arg1^+^ monocytes display inflammatory and profibrotic transcriptional programs and subsequently give rise to Ccr2^+^ macrophages that accumulate during the later phase of injury. By contrast, Mmp12^+^ macrophages increase after reversal of obstruction and are mainly associated with fibrosis resolution and tissue repair [22].

Homeostatic and repair-associated macrophages also comprise multiple transcriptional states. Mouse kidney-resident macrophages broadly express C1qa, C1qc, Cd81, and Ms4a7. An Mrc1^hi^ resident macrophage subset shows relatively high anti-inflammatory and wound-healing scores after ischemic injury. This subset expresses *Pf4*, *Fcrls*, and *Pdgfa* on day 1 after injury and *Igf1*, *Fcrls*, and *Timp2* on day 3. A separate resident macrophage subset is enriched for *Slc40a1* and shows increased expression of angiogenesis-associated genes, including *Igf1*, *Tnfaip2*, and *Vcam1*, after injury [19].

Under physiological conditions, renal macrophages clear cellular debris and immune complexes, provide immune surveillance, and contribute to tubulointerstitial homeostasis. Following kidney injury, resident macrophages and recruited monocyte-derived macrophages undergo stage-dependent functional reprogramming. During early AKI, inflammatory monocyte-derived macrophages can aggravate tubular and microvascular injury. As the initial injury subsides, macrophages participate in the clearance of necrotic material, resolution of inflammation, and tubular repair [23,24]. When repair fails, monocyte recruitment persists. During the AKI-to-CKD transition, macrophages progressively redistribute toward regions enriched in fibroblasts and ECM and acquire transcriptional programs associated with inflammatory regulation and matrix remodeling [20]. In established CKD, persistent injury maintains macrophage states with overlapping inflammatory, metabolic, and profibrotic programs, thereby contributing to chronic inflammation and fibrosis [20,25]. Because individual macrophage populations can either aggravate or limit tissue injury, their biological significance should be interpreted according to cellular origin, spatial location, disease stage, and functional behavior rather than inferred from a single marker.

### 2.2. Fibroblasts in Kidney Injury

In the normal kidney, fibroblasts are mainly located in the peritubular and perivascular interstitium. Collagen-reporter and lineage-tracing studies indicate that resident renal fibroblasts and pericyte-like cells are the major sources of collagen-producing cells and myofibroblasts after kidney injury, whereas the direct contribution of bone marrow-derived fibrocytes is relatively limited [26,27]. During kidney development, Foxd1-lineage mesenchymal progenitors give rise to adult CD73^+^ and PDGFRβ^+^ interstitial cells and pericytes. Following injury, these resident stromal populations expand and account for most α-SMA^+^ interstitial myofibroblasts [26]. Renal myofibroblasts have also been proposed to arise through epithelial–mesenchymal transition, endothelial–mesenchymal transition, or the recruitment and differentiation of bone marrow-derived cells. However, the contribution and mechanisms remain controversial [28,29]. Overall, current genetic lineage-tracing evidence more strongly supports resident renal stromal cells as the major source of myofibroblasts in experimental kidney fibrosis [26,30].

Renal fibroblasts and pericytes overlap in anatomical location, developmental origin, and the expression of markers such as PDGFRβ. When definitive lineage-tracing evidence is unavailable, the broader term “resident renal stromal cells” may therefore be used, with specific populations subsequently distinguished according to anatomical location and function. Under physiological conditions, fibroblasts participate in basal ECM turnover and provide structural support. A subset of interstitial fibroblasts produces erythropoietin (EPO) and can acquire myofibroblast characteristics after sustained injury, whereas pericytes contribute primarily to microvascular stability [26,31,32].

Reliable identification of renal fibroblasts requires a combination of markers. PDGFRA and DCN mark several fibroblast populations, while PDGFRβ is not lineage-specific as it is found in multiple interstitial and vascular cell types. After injury, COL1A1 and FN1 indicate ECM production, while α-SMA reflects contractility. POSTN and FAP are associated with activated or fibrotic fibroblasts [33,34]. However, these markers have limitations: α-SMA misses inflammatory or weakly contractile states; COL1A1 and FN1 may not represent contractile populations; vimentin is expressed by other mesenchymal cells and injured tubular cells; FSP-1 (S100A4) lacks specificity as it appears in inflammatory and bone marrow-derived cells. Thus, single markers are insufficient and should be used alongside other markers, anatomical location, and transcriptional profiles [35,36,37].

Single-cell and spatial transcriptomic analyses have further revealed the functional heterogeneity of conventional renal fibroblasts. Human fibrotic kidney tissue contains relatively quiescent fibroblasts and several myofibroblast states with different levels of ECM production, rather than a single homogeneous population of activated fibroblasts [34]. In mouse models of long-term unilateral IRI and UUO, three fibroblast clusters were identified: Secretory fibroblasts are enriched for *Col1a1* and *Fn1*, indicating active ECM production. Contractile fibroblasts express *Acta2* and *Myh11*, genes linked to muscle development and contraction. The migratory cluster is defined by transcriptomic enrichment for hematopoietic, immune-related, and neuron-projection programs, rather than by a direct migration assay [36]. A proinflammatory fibroblast population, termed CXCL-iFibro, has been characterized as an early state of myofibroblast differentiation in CKD [35]. Following kidney injury in aged mice, resident fibroblasts can differentiate into p75NTR^+^ fibroblasts producing CXCL13 or CCL19 and these cells participate in the formation and maturation of tertiary lymphoid tissues, indicating that the functions of renal fibroblasts extend beyond ECM production [38]. Collectively, renal fibroblast classification should integrate marker combinations, spatial location, and functional programs to distinguish inflammatory, ECM-secretory, contractile, and other injury-associated states.

Under homeostatic conditions, renal fibroblasts not only produce collagen but also participate in ECM turnover, microvascular support, and EPO production. During early AKI, resident fibroblasts become activated and enter the cell cycle before marked tubular epithelial proliferation occurs. This transient response may provide a provisional matrix and trophic support for tissue repair. Accordingly, the early appearance of α-SMA^+^ or PDGFRβ^+^ interstitial cells does not necessarily indicate irreversible fibrosis [39]. When repair proceeds successfully, some activated fibroblasts undergo apoptosis or decline in number, whereas others partially recover quiescent features. When repair fails, particularly in the medulla, fibroblast populations are more likely to retain myofibroblast-associated transcriptional programs and become less responsive to physiological signals that normally terminate fibroblast activation [40,41]. The AKI-to-CKD transition therefore involves not only an increase in fibroblast abundance but also a shift from transient repair-associated activation toward persistent inflammatory, contractile, and ECM-secretory states. In established CKD, inflammatory fibroblasts contribute to immune-cell recruitment, contractile fibroblasts participate in interstitial remodeling, and ECM-secreting myofibroblasts continuously produce collagen and fibronectin [35,36]. At the same time, pericyte detachment may impair peritubular capillary stability, whereas the conversion of EPO-producing fibroblasts into myofibroblast-like cells can reduce renal EPO production [31,32]. These stage-dependent changes reflect a functional transition of renal fibroblasts from early supporters of tissue repair to major effector cells in chronic kidney fibrosis.

Although macrophages and fibroblasts have distinct primary functions, they form a closely connected regulatory network within the injured renal microenvironment. The following sections focus on the intercellular communication between these populations, summarize the major bidirectional signaling pathways, and examine how this dynamic interaction regulates inflammation, tissue repair, and fibrosis, thereby providing a mechanistic basis for the identification of potential therapeutic targets.

## 3. The Signaling Pathways of Macrophage–Fibroblast Crosstalk

In a particular microenvironment, growth factors required by a cell can originate either from the same cell (autocrine) or from distinct cell types (paracrine). The resulting signal exchange establishes a cell circuit, within which factors secreted by one cell type may modulate the proliferation, activation, and/or growth factor output of the other [42,43]. From experimental studies, fibrotic cytokine “circuits” have been computationally modeled, where macrophages provide key growth factors that activate fibroblasts, and fibroblasts provide cytokines that drive macrophage polarization [43,44].

### 3.1. TGF-β Signaling

Transforming growth factor beta 1 (TGF-β) is a key cytokine involved in the regulation of cellular and tissue fibrosis. Canonical TGF-β signaling involves Smad2/3 phosphorylation and activation [45]. The non-canonical TGF-β/Smad route typically activates ERK, the mitogen-activated protein kinase (MAPK), PI3K/Akt, and Rho GTPase downstream in the absence of Smad activity [46,47]. TGF-β1 drives renal fibrosis by binding to TGF-βR2/TGF-βR1, activating Smad2/3 signaling, and upregulating fibrotic genes to promote fibroblast–myofibroblast transition.

Multiple studies have already confirmed that, in renal fibrosis, M2-polarized macrophages secrete TGF-β1, which constitutes a positive feedback loop that reinforces their own M2 polarization and drives macrophage-to-myofibroblast transition (MMT) via the Smad3 signaling pathway [48,49]. There is also research confirming that TGF-β signaling is a major factor determining the differential polarization of M2a and M2c. Under excessive TGF-β stimulation, M2a undergoes a process known as MMT, whereas moderate TGF-β stimulation favors the polarization of M2c phenotype macrophages [49]. Concurrently, this macrophage-derived TGF-β1 acts in a paracrine manner to activate resident fibroblasts thereby accelerating the progression of fibrosis [50]. In addition to directly activating the TGF-β signaling pathway, the activity of TGF-β is also subject to cross-regulation by multiple other signaling proteins, through which it mediates bidirectional crosstalk between the two cell types. In diabetic kidney disease, macrophage-derived Galectin-3 functions as a TGF-β1 signaling enhancer. Through direct binding to TGFBR2 on fibroblasts and inhibition of its ubiquitin-proteasome degradation, Galectin-3 markedly potentiates fibroblast responsiveness to TGFβ1, leading to amplified profibrotic signaling [51].

In addition to macrophage-derived TGF-β being capable of activating fibroblasts, another study has also confirmed that in hyperuricemia, activated Cxcl14^+^ fibroblasts secrete TGF-β, which acts on adjacent macrophages to sustain their Vim^+^ macrophage pro-fibrotic phenotype and enhance their pro-fibrotic cytokine production [52]. thereby creating a reciprocal activation loop that amplifies fibrotic signaling in the hyperuricemic renal microenvironment. It is evident that TGF-β signaling constitutes a bidirectional regulatory loop between macrophages and fibroblasts, and both cell types are capable of secreting TGF-β and influencing each other’s phenotype in a paracrine manner, thereby synergistically shaping the fibrotic microenvironment of the kidney.

### 3.2. Wnt/β-Catenin

The Wnt/β-catenin pathway, also known as the canonical Wnt signaling pathway, regulates cell proliferation, differentiation, and polarization during embryogenesis. While largely quiescent in the adult kidney, it is reactivated in various renal diseases [53,54,55]. Wnt ligands can act in an autocrine or paracrine manner. In the kidney, the most extensively studied cell types in this context are tubular epithelial cells (involved in regeneration, injury, and the process of epithelial–mesenchymal transition) [53,56,57,58], and macrophages (M2 polarization) [59]. Renal tubular epithelial cells are recognized as a key source of Wnt ligands in kidney fibrosis, and their derived Wnt-mediated regulation of macrophages and fibroblasts is well-documented. Meanwhile, the Wnt signaling interplay between macrophages and fibroblasts is increasingly attracting research attention.

Tian et al. found that myeloid-derived Wnts, mainly secreted by infiltrating macrophages, activate renal fibroblasts via β-catenin signaling to drive their proliferation, myofibroblast transition, and extracellular matrix deposition, thereby promoting kidney fibrosis. Meanwhile, these Wnt signals also induce macrophage polarization, with a stronger effect on M2 than M1, and mediate TGF-β1 and multiple fibrosis-related protein expression [60]. A study identified a proinflammatory fibroblast subset (CXCL-iFibro) by single-cell sequencing, which recruits monocytes and induces their differentiation into FOLR2^+^ macrophages at early fibrotic stages. These macrophages then promote fibroblast-to-myofibroblast transition via Wnt/β-catenin, establishing a reciprocal Wnt-dependent crosstalk, complementing Tian’s finding of macrophage-derived Wnts as the predominant initiator [35]. In fibroblasts, the maintenance of their activated state and pro-fibrotic phenotype is helped by autocrine and paracrine WNT. Activation of the WNT pathway induces pro-inflammatory activation of macrophages and polarization to the M2 phenotype, whereas macrophage-derived WNT affects fibroblast activation and proliferation [61].

### 3.3. PDGF Signaling

Platelet-derived growth factor (PDGF) serves as a master initiator of tissue repair and regeneration, orchestrating cellular proliferation, migration, and angiogenesis. However, aberrant activation of signaling cascade is extensively implicated in the pathogenesis of various malignancies, fibrotic disorders, and atherosclerotic cardiovascular diseases. The PDGF family encompasses multiple dimeric isoforms (PDGF-AA, -BB, -AB, -CC, and -DD), which exert their pleiotropic effects by engaging two cognate receptor tyrosine kinases, PDGFR-α and PDGFR-β. Upon ligand-induced dimerization and autophosphorylation, these receptors recruit and activate downstream signaling mediators, including PI3K and MAPK cascades, ultimately governing fundamental cellular outcomes such as proliferation, survival, and motility [62]. The vast majority of fibroblasts express both PDGFRα and PDGFRβ. In turn, macrophages can provide PDGF ligands that are critical for fibroblast survival [63,64].

In an early study of inflamed human kidney tissues, activated macrophages and platelets serve as primary sources of PDGF, whereas interstitial fibroblast-like cells acquire PDGF responsiveness through PDGFR-β expression. Notably, PDGFR-β^+^ fibroblast-like cells are consistently located in proximity to macrophage and T-lymphocyte aggregates. This spatial adjacency not only underscores a functional macrophage–fibroblast interplay but also furnishes direct morphological evidence for a paracrine PDGF axis linking the two cell types [65]. Zhou et al. demonstrated that macrophage-derived PDGF-B promotes fibroblast proliferation and survival through activation of PDGFR signaling. Concurrently, the PDGF-D/EGF autocrine loop in fibroblasts serves as an additional proliferative support mechanism [43]. Another study found that in the polycystic kidney disease (PKD) microenvironment, PDGF signaling predominantly originates from macrophage subpopulations, including the IL-1B^+^ M1-C1 and SELENOP^+^ M2-C1 subsets. Its ligands extensively target multiple fibroblast subtypes, including SYT1^+^ FIB1, IGF1^+^ FIB2, and ACTA2^+^ Myo myofibroblasts. This signaling cascade, acting in concert with pathways such as Wnt and Notch, constitutes an “inflammation-fibrosis” signaling network that drives the progression of renal fibrosis [66].

### 3.4. CSF-1/CSF-1R Signaling

In fibrosis, fibroblasts regulate macrophage recruitment and polarization by secreting chemokines. CSF-1 secreted by fibroblasts binds to the CSF-1R on the macrophage surface, providing signals for macrophage survival, proliferation, and transcriptional regulation. Fibroblast-derived CSF-1 activates CSF-1R on macrophages in a paracrine manner, thereby constituting the core signaling axis that maintains the homeostasis of tissue-resident macrophages [67].

Under stimulation of an inflammatory microenvironment, fibroblasts secreted more CSF1, which subsequently promoted the expression of macrophage marker in fibroblasts [68]. But further confirmation of fibroblast-specific CSF-1 deficiency and rigorous lineage tracing is needed. Zhou et al. showed that fibroblast-derived CSF1 drives macrophage proliferation/survival via CSF1R, while macrophage-derived PDGFB promotes fibroblast growth via PDGFR, forming a bidirectional cross-feeding circuit. To test whether this mutually dependent two-cell system can achieve stability, they co-cultured the two types at initial ratios varying by up to 2500-fold and observed convergence of both ratios and absolute numbers within two weeks. This stability is not trivial; simple reciprocal growth factor provision would generate positive feedback and uncontrolled expansion, implying additional regulatory mechanisms. Combining computational modeling with experimental validation, the study identified that environmental carrying capacity, negative feedback, and physical cell–cell contact are essential for circuit stability [43].

### 3.5. Metabolic Signaling

Although hemodynamic overload, inflammation, and other factors have long been recognized as drivers of kidney disease progression, metabolic dysfunction has come to the forefront as a central mechanism bridging cellular stress and irreversible tissue remodeling [69,70]. The term metabolic reprogramming encompasses injury-induced alterations across multiple interconnected domains, including fatty acid oxidation, glycolysis, mitochondrial respiration, amino acid and nucleotide metabolism, redox homeostasis, and metabolite-triggered signaling cascades [71,72].

Renal fibrosis stems from the concerted action of a multicellular microenvironment, wherein damaged tubular epithelial cells, activated fibroblasts, macrophages, endothelial cells, pericytes, and ECM components engage in bidirectional crosstalk, collectively dictating whether tissue injury resolves with repair or progresses toward a self-perpetuating fibrotic state [73,74]. Within this microenvironment, fibroblasts and immune cells exhibit greater metabolic plasticity, enabling rapid shifts in substrate utilization during repair or chronic inflammation. These differences establish cell-specific thresholds at which a compensatory metabolic response transitions into a fate-altering injury program.

During the transition from AKI to CKD, NF-κB transcriptionally activates HIF-1α, which drives glycolytic metabolic reprogramming in macrophages. This metabolic shift sustains a pro-inflammatory macrophage phenotype that actively promotes renal fibrosis [75]. In parallel, exogenous TNF-α and IL-1β are capable of potently stimulating renal fibroblasts to secrete large amounts of IL-6 and IL-8, thereby amplifying local inflammation and aggravating kidney injury. Notably, fibroblasts derived from fibrotic kidneys display a substantially heightened sensitivity to IL-1β challenge, with an approximately 3- to 4-fold greater inflammatory response relative to fibroblasts from normal kidneys [76]. It delineated a cooperative interplay between macrophage-driven metabolic reprogramming and fibroblast hyper-responsiveness. Spatial transcriptomics revealed specific colocalization of these two cell types in fibrotic regions, forming a “metabolic-immune niche.” Hyperuricemia-driven metabolic stress, particularly hypoxia-related reprogramming, initiates fibrotic remodeling. The resulting ECM deposition exacerbates local hypoxia, which enhances macrophage glycolysis and modulates fibroblast activation directly or indirectly [52].

The inflammatory cascade triggered by oxidative injury is not a process independent of metabolic pathways; rather, it represents a pathological event driven by metabolic reprogramming. This redox imbalance serves as an upstream instigator that initiates multiple macrophage–fibroblast crosstalk pathways. Oxidative stress activates AMPK in macrophages, driving their metabolic reprogramming toward fatty acid oxidation and promoting their differentiation into Arg1^+^MMP12^+^ pro-fibrotic cells. These cells secrete TWEAK, which induces tubular epithelial cells to produce PDGFB, subsequently activating fibroblasts and promoting excessive ECM deposition, thereby mediating fibrotic progression after IRI [77]. During the AKI-to-CKD transition, a distinct population of ECM-remodeling-associated macrophages (EAMs) emerges. These EAMs exhibit spatial dependency on fibroblasts primarily through the Igf1–Igf1r signaling axis and concurrently upregulate lipid metabolism-related genes, including Fabp5, Trem2, and Apoe. This observation suggests that macrophage–fibroblast crosstalk may involve coordinated regulation of ECM remodeling and lipid metabolic reprogramming [20]. Exogenous succinate activates the macrophage SUCNR1 receptor and triggers the downstream p-Akt/p-GSK3β/β-catenin/CTGF signaling cascade, which promotes M2 polarization and paracrine release of pro-fibrotic factors that induce fibroblast activation [78]. Collectively, macrophage metabolic reprogramming, acting through these interconnected pathways, cooperatively facilitates fibroblast activation and excessive ECM deposition, establishing itself as a critical pathogenic driver in macrophage–fibroblast crosstalk that cannot be overlooked.

### 3.6. Extracellular Vesicles

The fibrotic environment is rich in extracellular vesicles (EVs), nanoscale membrane-bound particles released by cells into the extracellular space, primarily classified into exosomes, macrovesicles, and apoptotic bodies, which differ in biogenesis, release pathways, size, and contents [79,80,81,82]. Moreover, EVs carry bioactive molecules including proteins, various RNAs, lipids, and metabolites, enabling stable and efficient transmission of complex signals and thereby effectively coordinating intercellular communication.

A study demonstrated that norepinephrine (NE)-treated macrophages promoted renal fibroblast activation through the transfer of Notch2 intracellular domain (N2ICD)-enriched EVs to fibroblasts [83]. The recent discovery of filopodial tip vesicles (FTVs) adds a novel dimension to our understanding of macrophage-mediated fibrosis. Under high-glucose conditions, macrophages release IL11-enriched FTVs, which directly drive fibroblast activation and differentiation. This high-glucose/M1-FTV-IL11 axis represents a previously unrecognized intercellular communication pathway capable of transmitting fibrotic cues with remarkable specificity [84]. One study demonstrated that fibroblasts stimulated with TGF-β1 differentiate into myofibroblasts, and the exosomes released by these activated myofibroblasts can be taken up by macrophages, promoting the co-expression of macrophage and myofibroblast markers, a process termed MMT. Notably, treatment with the exosome inhibitor GW4869 significantly abrogated this transition, confirming the essential role of exosomes in mediating this intercellular [85].

### 3.7. Mechanotransduction Signaling

A study has shown that fibroblast contractions can lure mechanosensitive and migratory macrophages through soft fibrous collagen matrices. Once recruited, among the macrophages, only the profibrotic subtype, but not the proinflammatory one, directly engages fibroblasts via the αvβ3 integrin-Piezo1 axis, rapidly elevating fibroblast cytosolic calcium and promoting NFAT1 and YAP nuclear translocation, thereby driving fibroblast activation. This process is particularly critical on soft matrices that alone suppress spontaneous fibroblast activation. In essence, the physical contact with profibrotic macrophages mechanically kick-starts fibroblast activation in an otherwise nonpermissive soft environment [86]. Numerous studies have shown that macrophages sense stiff ECM, such as that produced by contractile fibroblasts, through different mechanoperception mechanisms, which generally drive profibrotic macrophage phenotypes and secretory functions [87,88,89,90,91,92].

Peng et al. showed that macrophage-derived vitronectin accumulates in fibrotic kidneys and activates fibroblasts via αvβ3 integrin-FAK-AKT. It should be noted that the role of mechanical forces was not explored in this study. However, given that integrins serve as sensors for both ligand engagement and matrix rigidity, it is plausible that VTN signaling and mechanical cues converge to reinforce fibroblast activation [93]. A study demonstrates that myeloid-specific Piezo1 deletion protects against renal fibrosis by restraining macrophage infiltration and activation, primarily through suppression of the CCL2-CCR2 chemokine axis and Notch signaling, and the calcium-calpain proinflammatory cascade [94]. Although direct evidence for Piezo1-mediated regulation of renal fibroblasts remains lacking, in skin fibrosis, Piezo1 has been shown to directly drive fibroblast proliferation through a positive feedback loop involving the Wnt2/Wnt11-CCL24 pathway, and that silencing Piezo1 attenuates fibrotic progression [95]. Furthermore, in pulmonary fibrosis, pro-fibrotic macrophages engage in direct physical contact with fibroblasts via αvβ3 integrin, which activates fibroblast Piezo1 channels within seconds. This triggers calcium influx and subsequent YAP/TAZ nuclear translocation to initiate fibrogenic gene expression. More recently, Cadherin-11 was identified as a critical node integrating Piezo1 mechanosignaling with interleukin-6 signaling, thereby sustaining fibroblast activation and fibrotic progression [96,97]. Collectively, these observations raise the intriguing but still unresolved question of whether macrophages may similarly activate renal fibroblasts through Piezo1-dependent mechanotransduction—a hypothesis that warrants further experimental investigation.

In summary, macrophages drive and sustain fibroblast activation and renal fibrosis not only through paracrine signaling, but also via mechanical mechanisms, including direct physical contact and extracellular matrix remodeling. These insights provide a robust theoretical foundation for developing anti-fibrotic therapies that target intercellular mechanotransduction.

### 3.8. Other Signaling Pathways

In addition to the canonical signaling pathways discussed above, multiple other signaling pathways are also involved in regulating macrophage–fibroblast crosstalk, although relevant studies remain relatively limited. In this section, these less extensively investigated pathways are consolidated into a single subsection for collective discussion.

A study revealed a spatially and functionally heterogeneous landscape of macrophage–fibroblast crosstalk in lupus nephritis (LN). Spatially distinct macrophage subsets drive fibroblasts toward two functionally divergent pathological phenotypes: activated tissue-resident macrophages in the interstitium instruct fibroblasts to acquire a pro-inflammatory myofibroblast (Myofib1) phenotype, whereas infiltrating injury-associated macrophages in the glomeruli promote their differentiation toward a pro-fibrotic myofibroblast (Myofib2) fate. These two differentiation trajectories are mediated by ligand-receptor interactions including Spp1/integrins, Sema4/PlexinB, and NAMPT/INSR, which coordinately drive both inflammatory injury and excessive matrix deposition in LN [98]. Other macrophage-derived factors such as galectin-3, a beta-galactoside-binding lectin, promote renal fibrosis after UUO through the profibrotic activation of renal fibroblasts. Furthermore, the absence of Galectin-3 did not affect macrophage inflammatory cytokine secretion in response to IFN-γ/LPS stimulation, nor did it alter TGF-β expression or Smad2/3 phosphorylation levels in the obstructed kidney. These findings collectively indicate that the profibrotic action of Galectin-3 operates independently of both the classical inflammatory cascade and the canonical TGF-β/Smad signaling pathway [99]. Different from the previously mentioned mechanism in which Galectin-3 promotes renal fibrosis by directly enhancing TGF-β1 signaling [51]. The two studies are not contradictory: Henderson et al. showed that Galectin-3 promotes fibrosis without altering TGF-β expression or Smad2/3 phosphorylation, whereas Chen et al. demonstrated that Galectin-3 enhances TGFβ1 signaling by stabilizing TGFBR2 and Pro-TGFβ1. Galectin-3 may therefore act as an amplifier rather than an obligatory mediator of TGF-β signaling, though whether its role depends on disease model and microenvironment warrants further investigation. Additionally, spatial multi-omics revealed that Vim^+^ macrophages secrete TNF-α to activate NF-κB signaling in adjacent Cxcl14^+^ fibroblasts, driving fibroblast activation and renal fibrosis in hyperuricemia; activated fibroblasts in turn secrete TGF-β to sustain the pro-fibrotic phenotype of macrophages, forming a positive feedback loop [52].

Although the signaling pathways discussed above diverge in their molecular activation mechanisms, they are functionally interwoven and collectively orchestrate the progression of renal inflammation and fibrosis. The complexity of this network is first reflected in the establishment of bidirectional crosstalk circuits between macrophages and fibroblasts. For instance, macrophage-derived PDGF drives fibroblast proliferation, while fibroblast-derived CSF-1 supports macrophage survival, a reciprocal regulation that reinforces the fibrotic response. More critically, these pathways exhibit synergistic amplification effects across both spatial and temporal dimensions. Among them, the TGF-β/Smad and Wnt/β-catenin axes constitute a “sustained pro-fibrotic” signaling module, whereas CCL2/CCR2 and CSF-1/CSF-1R primarily deliver “inflammatory initiation/amplification” signals, collectively orchestrating the dynamic progression of renal injury. Ultimately, they converge on a common pathological endpoint: phenotypic polarization of macrophages, coupled with extensive transformation of fibroblasts into α-SMA^+^ myofibroblasts, which drives excessive deposition of ECM. Therefore, a comprehensive understanding of this regulatory network requires moving beyond the isolated examination of individual pathways and instead recognizing it as an integrated signaling hub that merges metabolic, immune, and matrix-remodeling cues. The collective output of this network substantially exceeds the arithmetic sum of its individual constituents. This conceptual framework further implies that effective anti-fibrotic strategies may necessitate simultaneous blockade of multiple critical nodes within this network to successfully disrupt the vicious cycle.

The principal signaling pathways and supporting experimental models are summarized in Table 1.

## 4. Macrophage–Fibroblast Crosstalk in Kidney Injury

### 4.1. Macrophage–Fibroblast Crosstalk in AKI: From Early Inflammation to Late Repair

AKI is characterized by a rapid decline in renal function over hours to days, resulting from diverse etiologies. While renal function may recover in the short term, persistent injury or incomplete repair can lead to the progression and transition to CKD. The hallmark pathological features of AKI include tubular epithelial cell injury, accompanied by renal interstitial edema and inflammation.

Macrophages do not assume a fixed role during different stages of AKI but rather exhibit a flexible switch between “pro-inflammatory injury” and “reparative healing.” Temporally, in the earliest phase, the primary task of infiltrating macrophages is to clear necrotic debris. However, as tissue injury exacerbates or in response to specific signals, macrophages undergo massive recruitment and infiltration, which in turn amplifies the local inflammatory response [100,101,102]. In IRI, cisplatin-induced, and sepsis-associated AKI models, Ly6C^hi^ monocytes infiltrate the kidney and differentiate into pro-inflammatory macrophages characterized by S100A8^+^S100A9^+^, IL-1β^hi^, TNF^hi^ or Saa3^hi^Ccl2^hi^ macrophages. These subsets exacerbate kidney injury through secretion of IL-1β, TNF-α and Saa3 [19,103]. Wang et al. revealed that injured proximal tubular cells recruit pro-inflammatory macrophages via integrin β1-rich EVs, which attract Fn1^+^ macrophages to exacerbate IRI-induced inflammation [104]. Intervention at this stage, targeting inflammation and pro-inflammatory macrophage polarization, has been shown to alleviate the severity of AKI and reduce the risk of subsequent fibrosis in mouse models. However, this process is extremely transient, as macrophages rapidly undergo a phenotypic switch toward the pro-reparative (M2) profile and release anti-inflammatory mediators (IL-10 and TGF-β), thereby limiting the inflammatory response [101,105]. M2 macrophages generate extracellular matrix. Dysregulated wound-healing macrophages play an important role in kidney fibrosis [100]. Studies have shown that stimulation of the cholinergic anti-inflammatory pathway suppresses TNF-α production while promoting macrophage polarization toward a protective M2 phenotype [106].

During AKI, early and transient activation of fibroblasts is critical for initiating kidney self-repair. Despite their limited abundance, fibroblasts are situated in close proximity to injured tubules, enabling them to rapidly respond to environmental cues and undergo activation upon local cellular regulation, thereby establishing themselves as one of the earliest responsive cell types in AKI. Vimentin^+^ fibroblasts are responsive as early as 1 h following ischemic AKI [39]. Once activated, fibroblasts produce ECM components such as collagens and laminins, which stabilize the tubular basement membrane, recruit growth factors, and modulate tensional homeostasis [107]. Concurrently, the ECM glycoprotein Nidogen-1 secreted by fibroblasts fosters a pro-repair microenvironment by engaging integrins and inducing Wnt signaling [108]. Moreover, fibroblasts release various metabolic modulators, including hepatocyte growth factor (HGF), to protect renal tubules, and during their transition to myofibroblasts, they produce retinoic acid to promote tubular regeneration [109,110,111]. After AKI, early activated fibroblasts exhibit divergent fates: some revert to quiescence, some are eliminated via programmed cell death, and others remain persistently activated, leading to aberrant repair and progression to fibrosis [112]. A study has shown that their fate is regulated by spatial localization: cortical fibroblasts tend toward quiescence, whereas medullary fibroblasts more often differentiate into myofibroblasts, driving the transition from AKI to CKD [41].

Crosstalk signaling between macrophages and fibroblasts is also present in AKI. A 2024 scRNA-seq study on AKI identified an Egr1-high fibroblast subpopulation (Egr1^+^ Fib-C1) as the most critical fibroblast subset linked to AKI. Egr1 is recognized as a ferroptosis-associated gene with a well-documented role in AKI pathogenesis. Meanwhile, a macrophage subset characterized by elevated Cxcl2 expression (Cxcl2^+^ Mac-C1) was defined as the key macrophage population in AKI, contributing to inflammation primarily through CXCL2 secretion. Intercellular communication analysis revealed multiple signaling interactions between Egr1^+^ Fib-C1 fibroblasts and Cxcl2^+^ Mac-C1 macrophages. The high CXCL2 production by Cxcl2^+^ Mac-C1 macrophages may induce a stress-responsive state in fibroblasts, or alternatively, activate CXCR2-expressing fibroblasts directly, thereby facilitating their migration, proliferation, and phenotypic shift toward a pro-inflammatory or pro-fibrotic state. In return, activated Egr1^+^ Fib-C1 fibroblasts may modulate macrophage behavior through chemokine regulation (CCL2 and CXCL1) or growth factor signaling [113]. Such crosstalk could further recruit circulating monocytes/macrophages to sites of injury, amplify local inflammation, or drive macrophages toward a pro-fibrotic polarization, collectively forming a self-perpetuating feedback loop, which drives the transition from AKI to CKD [114].

### 4.2. Macrophage–Fibroblast Crosstalk in CKD: Coexistence and Interaction of Phenotypes and Signaling Pathways

In the progression of primary CKD, persistent underlying causes, including metabolic abnormalities, long-standing hypertension, and ongoing immune activation, subject nephrons to sustained low-grade injury. This pattern is distinct from the acute and transient injury observed in AKI. The chronic injury microenvironment continuously releases chemokines such as CCL2, which recruit circulating monocytes to infiltrate the kidney and differentiate into macrophages. Unlike the transient M1-dominant pro-inflammatory phase followed by an orderly transition to the M2 repair phenotype seen in AKI [48], macrophages within CKD lesions maintain a mixed activation state over the long term. Single-cell sequencing studies have confirmed the presence of macrophage subpopulations exhibiting both pro-inflammatory and pro-repair features during CKD progression [115]. Macrophage populations within the same lesion simultaneously release pro-inflammatory cytokines (TNF-α, IL-1β) and pro-fibrotic factors (TGF-β), which sustain chronic low-grade inflammation while directly activating interstitial fibroblasts.

The crosstalk between macrophages and fibroblasts constitutes a central hub that drives CKD progression. As discussed in previous sections, macrophages activate the TGF-β/Smad and PDGF signaling axes in fibroblasts by secreting soluble factors such as TGF-β and PDGF, thereby promoting fibroblast proliferation and differentiation into myofibroblasts. Once activated, fibroblasts can sustain their own activation through autocrine TGF-β signaling. They also secrete CSF-1 and the chemokine CCL2, which reciprocally expand and maintain macrophage recruitment to the sites of injury. This process establishes a self-sustaining positive feedback loop that operates independently of the initial injury stimulation. In addition, aberrant activation of the Wnt/β-catenin signaling pathway during this crosstalk cooperates with TGF-β to drive extensive collagen synthesis by fibroblasts. Fibroblast-derived factors such as IL-6 stabilize the pro-inflammatory and pro-fibrotic phenotype of macrophages by inducing metabolic reprogramming, including enhanced glycolysis and fatty acid oxidation [61,116,117,118].

Notably, as CKD continues to progress, the accumulation of uremic toxins and local metabolic disturbances further intensify this cellular crosstalk. Concurrently, increased extracellular matrix stiffness during renal fibrosis amplifies the responsiveness of fibroblasts to TGF-β and enhances macrophage adhesion and activation through mechanical transduction pathways such as YAP/TAZ [116]. This process incorporates physical microenvironmental changes into the chemical interaction network. Ultimately, driven by the multidimensional interplay of paracrine signaling, metabolic reprogramming, and mechanical signals, macrophages continuously supply pro-fibrotic signals, while fibroblasts are persistently driven to synthesize and secrete large amounts of extracellular matrix components. These processes collectively accelerate renal interstitial fibrosis, leading to irreversible nephron loss and progressive decline in renal function, which constitutes one of the core mechanisms underlying the irreversible progression of CKD.

## 5. Targeted Therapy of Kidney Diseases

### 5.1. Established Clinical Therapies

#### 5.1.1. RAAS Inhibitors

Inhibition of the renin–angiotensin–aldosterone system (RAAS) remains a cornerstone of treatment for albuminuric CKD. The 2024 Kidney Disease: Improving Global Outcomes (KDIGO) guideline recommends an angiotensin-converting enzyme inhibitor (ACEI) or angiotensin II receptor blocker (ARB) for patients with CKD and moderately to severely increased albuminuria, with recommendations tailored to diabetes status. Treatment should be titrated to the highest approved dose that is tolerated [119]. In the REIN trial, ramipril reduced proteinuria and slowed the decline in glomerular filtration rate (GFR) in patients with severe proteinuric, non-diabetic nephropathy. The RENAAL trial subsequently showed that losartan reduced the risk of renal events, including doubling of serum creatinine and end-stage kidney disease, in patients with type 2 diabetes and nephropathy [120,121]. By lowering intraglomerular pressure and proteinuria, these agents reduce prolonged tubular exposure to excess filtered proteins and the associated inflammatory response, thereby attenuating upstream drivers of interstitial fibrosis.

Nonsteroidal mineralocorticoid receptor antagonists (MRAs) have further extended RAAS-based therapy. In FIDELIO-DKD, finerenone reduced the risk of a composite outcome comprising kidney failure, a sustained decline in estimated GFR (eGFR), or death from renal causes among patients with type 2 diabetes and CKD who were already receiving a maximally tolerated ACEI or ARB. The pooled FIDELITY analysis likewise demonstrated kidney and cardiovascular benefits across a broader range of CKD stages [122,123]. In ESAX-DN, esaxerenone reduced the urinary albumin-to-creatinine ratio (UACR) and increased the rate of albuminuria remission in patients receiving a renin–angiotensin system inhibitor [124]. However, esaxerenone is currently approved mainly for hypertension in Japan and has not been widely approved as a therapy that improves long-term kidney outcomes in CKD [125]. In clinical practice, additional treatment is best selected according to residual risk despite standard RAAS inhibition. For example, when UACR remains elevated and serum potassium permits, a nonsteroidal MRA may be considered in addition to an ACEI or ARB according to guideline recommendations and individual patient characteristics.

#### 5.1.2. SGLT2 Inhibitors

Preclinical studies have provided evidence for the antifibrotic effects of sodium–glucose cotransporter 2 (SGLT2) inhibitors. In rats subjected to 5/6 nephrectomy, empagliflozin improved kidney function and reduced glomerulosclerosis and interstitial fibrosis. It also altered the expression of genes associated with alternative activation or fibrosis, including Igf1, Trem2, Gpnmb, and Lgals3, in CD206^+^CD68^+^ macrophages and affected mitophagy and mechanistic target of rapamycin (mTOR) signaling [126]. These findings suggest that, in addition to reducing nephron workload, SGLT2 inhibitors may limit persistent profibrotic signaling by altering intrarenal macrophage states. In addition, emerging evidence indicates that SGLT2 inhibitors contribute to the attenuation of oxidative stress, which may retard the progression of renal fibrosis. SGLT2i have been shown to ameliorate DKD through the suppression of HIF-1α/HO-1-mediated redox imbalance and ferroptosis-driven peroxidative cascades [127]. Furthermore, integrated transcriptomic and metabolomic analyses have revealed that dapagliflozin inhibits NLRP3 inflammasome activation by promoting the production of itaconate, a TCA cycle-derived metabolite [128]. SGLT2 inhibition reduces renal oxidative stress, mitochondrial ROS production, and expression of apoptotic and fibrotic proteins while increasing autophagy in diabetic mice [129].

SGLT2 inhibitors have also become an important component of standard CKD treatment. In DAPA-CKD, dapagliflozin reduced the risk of the primary composite outcome—defined as a sustained decline in eGFR of at least 50%, end-stage kidney disease, or death from renal or cardiovascular causes—by 39%, with consistent benefit irrespective of diabetes status [130]. EMPA-KIDNEY enrolled 6609 patients with CKD at high risk of progression; empagliflozin reduced the risk of kidney disease progression or cardiovascular death by 28%, with consistent findings in participants with and without diabetes [131]. These clinical findings show that the kidney-protective effects of SGLT2 inhibition are not confined to patients with diabetes. Physiological and experimental studies suggest that these effects may involve restoration of tubuloglomerular feedback, reductions in glomerular hyperfiltration and intraglomerular pressure, and relief of tubular metabolic stress. These mechanisms, however, were not direct end points of DAPA-CKD or EMPA-KIDNEY.

### 5.2. Targeted Intervention in Signaling Pathways

#### 5.2.1. TGF-β/Smad Signaling

The TGF-β/Smad pathway links macrophage-derived paracrine signals, fibroblast activation, and ECM synthesis and is a central profibrotic pathway in the kidney.

Clinical studies have examined more selective approaches to TGF-β-associated fibrogenesis. Pirfenidone has broad antifibrotic activity, and experimental studies indicate that it can affect TGF-β-related signaling, inflammation, and matrix production. In a randomized trial of 77 patients with diabetic nephropathy, the 1200-mg/day group showed a signal of improved eGFR after 1 year [132]. In an open-label study of 21 patients with FSGS, the rate of eGFR decline among the 18 participants who completed treatment was approximately 25% lower than during the baseline period [133]. Kidney-directed inhibition may improve the selectivity of TGF-β-targeted therapy. In mice with UUO, a bispecific antibody that recognized the extra domain A (EDA) of fibronectin and neutralized TGF-β preferentially accumulated in fibrotic kidneys and reduced collagen deposition and the expression of Col1a1, FnEDA, and Timp1 [134]. This study shows that injury-associated ECM components can serve as molecular addresses that concentrate therapy within diseased kidneys and potentially limit exposure in other tissues. Future studies may examine whether this type of targeted delivery can be combined with inhibition of downstream mediators such as CTGF or Smad3. Any advantage over conventional administration will require confirmation in models that more closely reproduce clinical CKD and, ultimately, in human studies.

Connective tissue growth factor (CTGF/CCN2), an important downstream effector of TGF-β, is associated with fibroblast activation and matrix deposition and has also been investigated therapeutically. In a phase 1 study of 24 patients with diabetes and microalbuminuria, the anti-CTGF antibody FG-3019 was well tolerated, and mean UACR decreased from 48 mg/g at baseline to 20 mg/g on day 56 [135]. Given the small sample size, this study provides early clinical support for further investigation of downstream TGF-β targets rather than definitive evidence of kidney protection.

#### 5.2.2. CCL2/CCR2 Signaling

The CCL2/CCR2 axis promotes the recruitment of inflammatory monocytes to the injured kidney and links persistent inflammation to kidney fibrosis. In patients with type 2 diabetes and nephropathy, the CCR2 inhibitor CCX140-B reduced residual albuminuria, with the 5-mg dose showing the most consistent effect [136]. Treatment with the CCL2 inhibitor emapticap pegol was also associated with a downward trend in UACR. In the primary intention-to-treat analysis, however, the adjusted between-group difference at week 12 was not statistically significant, although a sustained decline remained evident during the post-treatment observation period [137]. Its potential role during active inflammation or in other primary and secondary kidney diseases requires trials designed specifically for those settings.

Both CCX140-B and emapticap pegol remain investigational. Existing clinical studies have primarily used changes in UACR as efficacy measures and have not established kidney protection through long-term outcomes such as sustained eGFR decline, kidney failure, or dialysis. Future trials should prioritize patients with evidence of ongoing monocyte recruitment and inflammatory activity and should determine whether changes in the chemokine pathway are associated with long-term kidney outcomes.

#### 5.2.3. CSF-1/CSF-1R Signaling

CSF-1/CSF-1R signaling regulates monocyte–macrophage survival, expansion, and differentiation, but its effects may differ across disease stages. In mice with IRI, the CSF-1R inhibitor GW2580 reduced the accumulation of Ly6C^+^ macrophages and attenuated both acute injury and subsequent interstitial fibrosis [138]. During recovery, however, CSF-1 signaling promotes the expansion of intrarenal macrophages and dendritic cells and supports tubular regeneration [24]. Single-cell analysis has further identified both Arg1^+^ profibrotic monocytes and Mmp12^+^ macrophage states associated with matrix degradation and remodeling during disease progression and regression [22]. Prolonged, broad macrophage depletion is therefore unlikely to be an optimal strategy. A more rational approach would be stage-specific: transiently restricting monocyte entry during marked recruitment while preserving myeloid populations that support tissue recovery during the repair phase. Urinary chemokines, circulating CCR2^+^ monocytes, and kidney injury markers could serve as candidate measures for patient stratification and treatment adjustment in future studies.

#### 5.2.4. WNT/β-Catenin Signaling

WNT signaling is an important component of macrophage–fibroblast crosstalk. In experimental kidney fibrosis, myeloid-derived Wnts also promote macrophage and fibroblast activation, whereas genetic blockade of Wnt ligand secretion in myeloid cells reduces inflammation and collagen deposition [60]. In addition, ICG-001, which disrupts the interaction between β-catenin and CREB-binding protein (CBP), reduced α-smooth muscle actin (α-SMA), collagen, and fibronectin expression in UUO. Treatment initiated on day 3 after obstruction still limited further fibrotic progression [139].

The effect of WNT-directed therapy may depend on treatment timing. In mice with ischemic AKI, sequential administration of a Wnt agonist to promote early repair followed by an antagonist to limit persistent signaling supported acute tissue recovery while reducing later fibrosis [111]. These findings indicate that a transient WNT response after injury and sustained WNT/β-catenin activation may have different consequences, making temporally staged modulation a useful direction for further study. Future clinical studies could examine the interval from injury, the pattern of eGFR recovery, and urinary or tissue indicators of WNT pathway activity as potential guides to treatment timing. At present, however, none of these measures is an established clinical threshold for switching therapy.

#### 5.2.5. PDGF/PDGFR Signaling

PDGF/PDGFR signaling promotes the proliferation and migration of pericyte-like interstitial cells and their activation into myofibroblasts. In models of obstructive and post-ischemic kidney fibrosis, increased PDGFR-α/β expression coincided with pericyte-to-myofibroblast transition and ECM deposition, whereas pathway inhibition reduced activated interstitial cells and fibrosis [140]. These findings support the therapeutic potential of PDGFR inhibition during phases of kidney fibrosis characterized by active interstitial cell proliferation. Whether the abundance of PDGFR^+^ interstitial cells can guide patient selection or predict treatment response will require histological studies and prospective trials.

In mouse models of autosomal dominant polycystic kidney disease (ADPKD), Nintedanib reduced cyst growth and attenuated fibrosis in Pkd1RC/RC mice, although these effects were not fully reproduced in a second Pkd1 model [141]. Diarrhea and elevated liver enzyme levels, together with VEGFR inhibition-associated risks such as bleeding, arterial thromboembolism, nephrotic-range proteinuria, and glomerular microangiopathy, require particular attention in studies involving kidney disease. Nintedanib remains a candidate for further study, but clinical efficacy in CKD has not been established. Future work could focus on patients or models with active interstitial cell expansion and explore kidney-targeted delivery or shorter treatment courses to reduce systemic effects related to VEGFR inhibition.

### 5.3. Cell-Targeted Strategies and Stage-Based Therapy

#### 5.3.1. Functional Reprogramming of Macrophages

Macrophage-directed therapy is shifting from indiscriminate cell depletion toward modulation of specific macrophage functions. Arg1^+^ profibrotic monocytes, CCR2^+^ recruited monocytes/macrophages, and Mmp12^+^ macrophage subsets involved in matrix degradation and remodeling can coexist during the progression and regression of kidney injury [22]. The traditional M1/M2 framework is therefore insufficient to guide treatment directly. Current evidence instead supports treating persistent recruitment and preserving reparative functions as distinct therapeutic objectives, rather than targeting all F4/80^+^ or CD68^+^ cells for depletion. Empagliflozin can modify fibrosis-associated gene expression in CD206^+^CD68^+^ macrophages [126], suggesting that established therapies may exert part of their antifibrotic effect by changing macrophage states. Future studies could use kidney-targeted lipid nanoparticles or antibody-conjugated carriers to deliver agents against targets such as CCR2 or SPP1 more selectively to relevant cell populations.

#### 5.3.2. Kidney- and Cell-Directed Delivery

A central limitation of systemic treatment is the difficulty of confining drug exposure to the relevant kidney cell population. An LRG1-targeted Nintedanib delivery system increased drug accumulation in fibrotic kidneys and reduced ECM-related expression in experimental kidney fibrosis [142]. The FnEDA–TGF-β bispecific antibody likewise showed that injury-associated ECM can provide a molecular address for delivery to fibrotic kidneys [134]. These animal studies suggest that altering drug biodistribution may strengthen local activity while limiting exposure in other tissues. Whether targeted delivery improves both efficacy and safety will require additional pharmacokinetic, toxicological, and clinical studies.

#### 5.3.3. Therapeutic Windows and Treatment Sequencing

Current preclinical evidence supports a stage-specific therapeutic framework that remains to be validated, with different priorities during early AKI, the repair-to-transition phase, and active CKD. Animal studies show that early fibroblast activation and tubular sonic hedgehog signaling contribute to kidney repair after injury [39]. During early AKI, the first priorities are to maintain perfusion and control excessive inflammation while preserving signals required for epithelial regeneration. During the transition from AKI to CKD, CCR2-dependent cell recruitment and persistent WNT or TGF-β signaling may become more relevant targets. In active CKD, fibroblast expansion, ECM production, and matrix cross-linking may warrant greater attention. This staged framework is derived mainly from animal studies and early clinical evidence and does not yet constitute an established clinical pathway.

#### 5.3.4. Kidney Organoids and Regenerative Models

Kidney organoids derived from pluripotent stem cells can form glomerular- and tubular-like structures. After transplantation beneath the kidney capsule of mice, organoids acquire host-derived vasculature and show further glomerular and tubular maturation [143]. At present, their most practical applications remain disease modeling and drug screening rather than replacement of an intact kidney. If such models can reproducibly capture patient-specific fibrotic features, they may support preclinical patient stratification and combination-drug screening and provide platforms for assessing maturity and safety in future tissue-replacement research. Even transplanting lab-grown artificial kidneys into patients could enable them to long-term and stably compensate for or even completely replace the original kidneys’ physiological functioning.

## 6. Discussion

In this review, we systematically survey the origins, physiological functions, and pathological roles of renal fibroblasts and macrophages. On this basis, we elaborate on the crosstalk mechanisms between these two cell types mediated by signaling pathways. We further discuss their phenotypic dynamics and functional transitions during the course of AKI and CKD. Finally, in view of the functional heterogeneity of fibroblasts and macrophages across kidney injury, we summarize current advances in targeted therapeutic strategies and explore their potential clinical applications.

Macrophages and fibroblasts play critical roles in both the maintenance of renal physiology and the pathogenesis of kidney injury, engaging in complex crosstalk that involves multiple signaling pathways and o organs. As above, this crosstalk is mediated through diverse mechanisms, including various molecular signaling pathways, release of EVs, regulation of the ECM microenvironment, and direct cell–cell contact. Substantial evidence indicates that macrophage–fibroblast communication also occurs in other tissues and organs, such as the heart, lung, liver, and tumors. Importantly, the essence of this interaction does not lie in a simplistic designation of either cell type as “pro-fibrotic” or “pro-inflammatory”; rather, it is characterized by a dynamic feedback loop involving both cell phenotypes and the ECM. Accordingly, cell phenotypes should not be regarded as static. Both macrophages and fibroblasts exhibit continuously shifting pro-inflammatory and pro-fibrotic states. Moreover, macrophages cannot be simply classified into M1/M2, with distinct phenotypic states even coexisting within the same cell population.

Beyond the complex crosstalk between macrophages and fibroblasts, accumulating evidence indicates that macrophage-to-myofibroblast transition directly contributes to fibrosis. Conversely, early studies demonstrated that co-expression of PU.1 with C/EBPα or C/EBPβ reprograms mouse embryonic fibroblasts (MEFs) or dermal fibroblasts into macrophage-like cells, which acquire macrophage markers, phagocytic capacity, partial inflammatory responsiveness, and CSF-1-dependent growth. More recently, optimization of conventional induced pluripotent stem cell (iPSC) differentiation has revealed that expression of c-Myc alone suffices to directly reprogram MEFs into functional macrophages (iMacs) in vitro, which display a distinct M1-like (pro-inflammatory) phenotype alongside phagocytic activity and antitumor effector functions. Additionally, fibroblasts have been reported to express macrophage markers under inflammatory conditions, though this observation lacks functional characterization and definitive in vivo genetic lineage tracing [68]. Thus, whether fibroblasts could transdifferentiate into functional macrophages in vivo remains an open question. Addressing this will require a comprehensive chain of evidence, encompassing cell-specific genetic manipulation, spatial protein validation, in vivo functional recovery assays, and long-term lineage tracing, a goal that remains considerably distant at present.

The present work offers an initial investigation into macrophage–fibroblast interactions in AKI and CKD. Given the substantial overlap between this content and the preceding section on signaling pathways, we have not expanded upon it in detail. Furthermore, due to the limited number of currently available reports, this review does not offer a systematic classification or summary of common renal diseases, such as diabetic nephropathy, hypertensive kidney injury, and lupus nephritis, based on clinical etiology or pathological features. These aspects await further elaboration in future studies.

With the advancement of scRNA-seq, spatial transcriptomics, and lineage-tracing technologies, the phenotypic heterogeneity of macrophages and fibroblasts, and their interaction networks in different renal diseases are expected to be analyzed in more detail. Especially for common kidney diseases, there is an urgent need for more high-quality basic and clinical studies to clarify the specific contributions of cell–cell crosstalk at distinct pathological stages and to provide a theoretical basis for targeted intervention strategies. In summary, research in this field is currently in exploration, with substantial space for future investigation and translational potential.

Finally, as a narrative review, this article inevitably carries the authors’ subjective selection and integration of the literature and does not cover all the published data. Therefore, the discussion presented here should not be interpreted as a systematic summary of renal fibrosis. With progress in single-cell multi-omics and in vivo lineage tracing, we expect the macrophage–fibroblast crosstalk to undergo further validation and refinement.

## Figures and Tables

**Table 1:** Signaling pathways of macrophage–fibroblast crosstalk.

Signaling Pathway	Signaling Cell	Responding Cell	Function (Macrophage–Fibroblast Crosstalk)	Model	Source
**TGF-β**	M2 macrophage	Fibroblast	M2 macrophage-derived Galectin-3 potentiates TGF-β1 signaling by stabilizing both TGFBR2 and Pro-TGF-β1, consequently driving fibroblast activation toward a pro-fibrotic myofibroblast phenotype with increased α-SMA expression.	DKD mouse models, DKD patient samples, and in vitro cell experiments.	[51]
	Cxcl14^+^ fibroblasts	Vim^+^ macro-phage	TGF-β secreted by Cxcl14^+^ fibroblasts sustains Vim^+^ macrophage pro-fibrotic phenotype and enhances their pro-fibrotic cytokine production.	Hyperuricemic mouse model	[52]
**Wnt/** **β-catenin**	M1 and M2 macrophage	Fibroblast	Polarized BMDMs induced multiple Wnt ligands. Myeloid-specific ablation of Wls blocked macrophage Wnt secretion and attenuated fibroblast activation. Conditioned medium from Wls-deficient BMDMs inhibits fibroblast proliferation and activation	UUO mouse model and in vitro experiments.	[60]
**PDGF**	FOLR2^+^ macrophages	CXCL-iFibro	FOLR2^+^ macrophage-derived Wnt activates β-catenin in CXCL-iFibro fibroblasts, promoting their transition to ECM-producing myofibroblasts.	CKD patients as well as in vitro experiments.	[35]
	Macrophage	Fibroblast	Activated macrophages secreted PDGF, and interstitial fibroblasts upregulated PDGFR-β to gain PDGF responsiveness, leading to their proliferation.	Renal tissue from human chronic rejection and glomerulonephritis	[65]
	Macrophage	Fibroblast	Macrophage-derived PDGF-B promoted fibroblast proliferation and survival through activation of PDGFR signaling.	In vitro experiments	[43]
	Macrophage	Fibroblast	Macrophage-derived PDGF targets fibroblasts and synergizes with Wnt/Notch signaling to drive kidney fibrosis	Renal tissue specimens from ADPKD patients	[66]
**CSF-1/** **CSF-1R**	Fibroblast	Macrophage	Fibroblast-derived CSF1 drives macrophage proliferation/survival via CSF1R	In vitro experiments	[43]
**Extracellular** **vesicles**	Macrophage	Fibroblast	Norepinephrine (NE)-treated macrophages promoted renal fibroblast activation through the transfer of Notch2 intracellular domain (N2ICD)-enriched EVs to fibroblasts	Npx-IRI mice/repeated low-dose cisplatin regimen and in vitro experiments	[83]
	M1 macrophage	Fibroblast	Under high-glucose conditions, macrophages release IL11-enriched FTVs, which directly drive fibroblast activation and differentiation.	STZ-diabetic mouse model, DN patient biopsies, and in vitro experiments	[84]
	TGF-β1- stimulated fibroblasts	Macrophage	Myofibroblast-derived exosomes (from TGF-β1-stimulated fibroblasts) are taken up by macrophages, promoting macrophage-to-myofibroblast transition	Folic acid (FA)-induced mouse model and in vitro experiments.	[85]
**Mechanotransduction**	Macrophage	Fibroblast	Macrophage-derived VTN accumulates in fibrotic kidneys and activates fibroblasts via αvβ3 integrin-FAK-AKT	Unilateral IRI mice and in vitro experiments	[93]
**Other signaling pathways**	Tissue-resident and infiltrating injury macrophages	Myofib1 and Myofib2	Activated tissue-resident macrophages in the interstitium instruct fibroblasts to acquire a pro-inflammatory myofibroblast (Myofib1) phenotype, whereas infiltrating injury-associated macrophages in the glomeruli promote their differentiation toward a pro-fibrotic myofibroblast (Myofib2) fate	Lupus nephritis renal biopsy specimen	[98]
	Macrophage	Fibroblast	Macrophage-derived factor, galectin-3, promotes renal fibrosis after UUO through the profibrotic activation of renal fibroblasts	UUO mice model	[99]
	Vim^+^ macrophage	Cxcl14^+^ fibroblasts	Spatial multi-omics revealed that Vim^+^ macrophages secrete TNF-α to activate NF-κB signaling in adjacent Cxcl1^+^ fibroblasts, driving fibroblast activation and renal fibrosis in hyperuricemia	Hyperuricemic mouse model	[52]
**Metabolic** **signaling**	M1 macrophage	Fibroblast	NF-κB activates HIF-1α to drive glycolytic reprogramming of macrophages into a pro-inflammatory phenotype, promoting cytokine secretion. TNF-α and IL-1β stimulate fibroblasts to release IL-6 and IL-8, amplifying inflammation and kidney injury.	IRI model, UUO model and in vitro cell experiments.	[75,76]
	Macrophage	Fibroblast	Hyperuricemia-driven metabolic stress, particularly reprogramming associated with hypoxia, enhances macrophage glycolysis and directly or indirectly regulates fibroblast activation.	Hyperuricemic mouse model	[52]
	macrophage	Fibroblast	Oxidative stress activates macrophage AMPK signaling, driving FAO-oriented metabolic reprogramming and Arg1^+^MMP12^+^ pro-fibrotic differentiation, whose secreted TWEAK induces tubular PDGFB production, thereby activating fibroblasts and promoting ECM overdeposition.	IRI mouse model	[77]
	EAMs	Fibroblast	EAMs exhibit spatial dependence on fibroblasts via Igf1–Igf1r signaling and upregulate Fabp5, Trem2, and Apoe, indicating coordinated regulation of ECM remodeling and lipid metabolic reprogramming.	Unilateral IRI-induced AKI-to-CKD mouse model	[20]
	M2 Macrophage	Fibroblast	Exogenous succinate activates macrophage SUCNR1, triggering p-Akt/p-GSK3β/β-catenin/CTGF signaling, M2 polarization, and paracrine release of pro-fibrotic factors that induce fibroblast activation.	C57/BL6 mice fed with 4% succinate in drinking water for 12 weeks	[78]

## Data Availability

No new data were created or analyzed in this study. Data sharing is not applicable to this article.
